# Risk and Protective Factors Associated with Non-Suicidal Self-Injury in Sexual and Gender Minority Individuals: A Scoping Review

**DOI:** 10.3390/ejihpe16070086

**Published:** 2026-06-25

**Authors:** Filipa Gomes, Carol Coelho, Daniela Fumega, Bárbara C. Machado, Sónia Gonçalves

**Affiliations:** 1 Centre of Research in Psychology (CiPsi), School of Psychology, University of Minho, 4704-553 Braga, Portugal; id11297@alunos.uminho.pt (C.C.); pg53545@alunos.uminho.pt (D.F.); sgoncalves@psi.uminho.pt (S.G.); 2 CEDH—Research Centre for Human Development, Faculdade de Educação e Psicologia, Universidade Católica Portuguesa, 4169-005 Porto, Portugal; bcmachado@ucp.pt

**Keywords:** minority stress model, non-suicidal self-injury, protective factors, risk factors, sexual and gender minorities

## Abstract

Non-suicidal self-injury (NSSI) is a significant public health concern, with disproportionately higher prevalence among sexual and gender minority (SGM) populations compared to cisgender heterosexual individuals. While prior research has examined NSSI and related outcomes in SGM groups, evidence on specific risk and protective factors remains limited. This scoping review aimed to systematically map and synthesize risk and protective factors associated with NSSI in SGM populations. A systematic search was conducted in PubMed, Scopus, and Web of Science up to 2 February 2026. A total of 43 studies were included, the majority of which were conducted in the United States and employed cross-sectional designs. Data were charted and synthesized using a minority stress-informed socioecological framework. Findings indicate that NSSI is consistently associated with the co-occurrence of minority stress processes and intrapersonal vulnerabilities. Additional risk factors were identified across family, peer, and community domains. Protective factors were less frequently examined but included social support, family connectedness, school safety, and adaptive coping strategies. Overall, the findings suggest that NSSI among SGM populations is best understood as the result of interacting risk processes across multiple ecological levels. These results support a minority stress-informed, multi-level conceptualization of NSSI in SGM individuals and highlight the need for longitudinal research and greater focus on protective factors.

## 1. Introduction

Non-suicidal self-injury (NSSI) is defined as the deliberate and direct destruction of body tissue without suicidal intent (Nock & Favazza, 2009). Common examples include cutting, burning, scratching, or hitting oneself (Katz-Wise et al., 2018). NSSI has been identified as a significant public health concern across adolescents, community samples, and clinical populations (Baer et al., 2020). Epidemiological evidence from nonclinical samples suggests that NSSI is relatively common across developmental stages, with pooled prevalence estimates of 17.2% among adolescents, 13.4% among young adults, and 5.5% among adults (Ross-Reed et al., 2019). The adverse clinical correlates of NSSI have been widely documented. Longitudinal research in adolescents indicates that NSSI is prospectively associated with depressive symptoms, hopelessness, and emotional dysregulation, while depressive symptoms may also predict subsequent NSSI, highlighting the reciprocal nature of these associations over time (Dunlop et al., 2022; Fraser et al., 2018). NSSI has also been consistently associated with suicidal ideation and suicide attempts, underscoring its relevance as an indicator of elevated suicide risk (Katz-Wise et al., 2018).

Meta-analytic evidence indicates that sexual and gender minority (SGM) individuals—referring to individuals whose sexual orientation and/or gender identity differ from heterosexual and cisgender norms—experience higher rates of NSSI compared with cisgender heterosexual populations (Li et al., 2019). This evidence further demonstrates marked disparities in NSSI prevalence, with lifetime estimates ranging from approximately 30% among sexual minority individuals to 47% among gender minority individuals, compared with approximately 14.6% among heterosexual and/or cisgender peers (Li et al., 2019). These disparities are frequently explained through the minority stress framework, which proposes that stigma, discrimination, and victimization related to sexual orientation and gender identity create chronic stressors that negatively affect mental health (Meyer, 2003). Consistent with this framework, SGM populations experience elevated levels of psychological distress, depression, and suicidal behaviors compared with heterosexual and cisgender individuals (Peters et al., 2020). Within this perspective, NSSI risk may be understood as arising from interrelated influences operating across individual, interpersonal, and structural levels, consistent with socioecological models of health (Bird et al., 2024).

In this regard, a growing number of studies have examined factors associated with NSSI in SGM populations. These studies have identified a range of potential correlates, including psychological distress, experiences of discrimination or victimization, exposure to violence, and limited social support (Benau et al., 2017; Taliaferro et al., 2024). At the same time, protective factors such as supportive family relationships, social connectedness, and affirming environments have also been highlighted in the literature (Benau et al., 2017). However, the existing evidence remains heterogeneous, with studies varying in the factors examined and the measures used to assess NSSI and related behaviors, which limits the ability to synthesize findings across studies (Benau et al., 2017).

Although elevated risk of NSSI among SGM populations is well-documented, existing evidence syntheses have largely focused on prevalence estimates and broad psychosocial correlates. Meta-analyses, such as those by Batejan et al. (2015) and Liu et al. (2019), have provided important quantitative evidence showing that SGM individuals report higher odds of NSSI than heterosexual and cisgender individuals. However, some reviews have examined NSSI alongside suicidal ideation, suicide attempts, or broader self-harm outcomes, which may limit understanding of the mechanisms and potentially modifiable factors that are specific to NSSI. Furthermore, while systematic reviews like Rogers and Taliaferro (2020) have mapped recent developments in the field, there remains limited synthesis of both risk and protective factors within an integrated socioecological framework.

The present scoping review addresses this gap by moving beyond prevalence estimates to systematically map multi-level risk and protective factors associated with NSSI in SGM individuals. Rather than prioritizing pooled statistical associations, this study employs a minority stress-informed socioecological framework to organize risk and protection across individual, interpersonal, community, and structural levels (Meyer, 2003; Liang & Chen, 2024). By explicitly separating NSSI from suicidal thoughts and behaviors and focusing on the interplay between distal minority stressors and proximal psychological vulnerabilities, this review provides a more nuanced account of how risk may accumulate and interact across ecological contexts (Fraser et al., 2018; Ramsay-Patel et al., 2026). Consequently, this work may inform the development of targeted, strengths-based prevention protocols that incorporate resilience-promoting strategies alongside traditional risk-reduction efforts.

## 2. Materials and Methods

### 2.1. Protocol and Registration

The review was conducted in accordance with the PRISMA-ScR guidelines (Page et al., 2021) and was prospectively registered with the Open Science Framework (OSF) in April 2026. While a separate, standalone protocol document was not published as a preprint, the OSF registration record provided a comprehensive account of the research questions, electronic database search strategies, and inclusion/exclusion criteria. This prospective registration served as the primary methodological reference for the review, ensuring transparency throughout the study selection and data-charting processes. PRISMA-ScR checklist can be found at Table S1.

### 2.2. Search Strategy and Selection Criteria

This review followed the methodological framework for scoping studies comprising five stages: (1) identifying the research question; (2) identifying relevant studies; (3) study selection; (4) charting data; and (5) collating, summarizing, and reporting results (Arksey & O’Malley, 2005). A systematic search was conducted in three electronic databases: PubMed, Scopus, and Web of Science, with the final search performed on 2 February 2026. Search strategies were tailored to each database and combined three core conceptual domains: (1) sexual and gender minority populations; (2) NSSI; and (3) risk and/or protective factors (e.g., stigma, discrimination, marginalization, family, school, bullying, healthcare, policy, community, social support). Controlled vocabulary and free-text terms (e.g., MeSH terms) were used when appropriate. The complete search strategies for each database are presented in Table A1.

Studies were eligible for inclusion if they met pre-defined eligibility criteria regarding population, outcome, study design, publication type, language, and availability. Concerning the population, eligible studies had to focus on adolescents (≤18 years) or adults identifying as sexual and/or gender minorities (SGM), including but not limited to lesbian, gay, bisexual, transgender, non-binary, and queer identities. The primary outcome of interest was NSSI. Accordingly, studies were required to assess NSSI as a distinct construct, including its history, frequency, characteristics, correlates, or functions, or to report data in which non-suicidal self-injury was analysed separately from suicidal behaviors or broader self-harm outcomes. We considered primary research using quantitative, qualitative, or mixed methods designs, as well as formal evidence syntheses. To ensure a minimum level of reporting transparency, inclusion was restricted to peer-reviewed publications, thereby excluding gray literature such as dissertations, conference proceedings, preprints, and technical reports. Furthermore, the search was limited to studies published in English for which the full text could be retrieved through institutional access, open-access sources, or direct contact with authors. Finally, no lower-bound restriction was applied to the year of publication, and all relevant studies published up to the final search date of 2 February 2026 were considered eligible.

**Figure 1:**
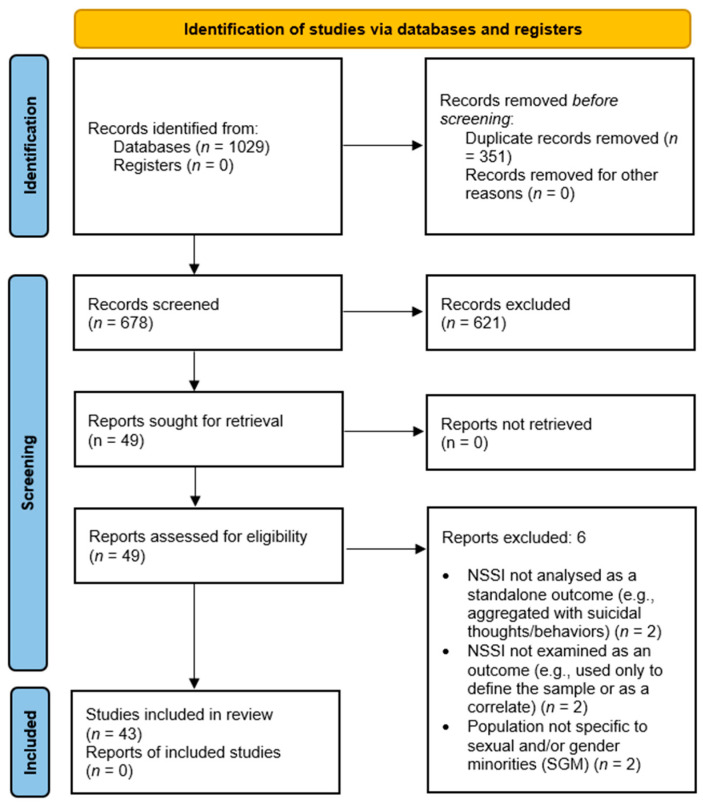
PRISMA Flowchart.

All records were imported into Rayyan (Ouzzani et al., 2016) for reference management and screening. After retrieval, duplicates were automatically identified and removed. The first step of screening was based on titles, keywords, and abstracts. The second step included a full-text review of the remaining articles, with eligibility assessed against the inclusion criteria. All screening stages were conducted independently by two reviewers, and any discrepancies were resolved through discussion with a third reviewer. Reasons for full-text exclusion are reported in Figure 1.

### 2.3. Data Extraction

Data were extracted using a standardized charting form developed iteratively, an approach recommended for scoping reviews to refine and support consistency in data charting (Levac et al., 2010). Extracted variables included author(s), year of publication, country and study context, study design, sample size, age range, SGM subgroup, operationalization and measurement of NSSI, theoretical framework (when specified), examined risk and protective factors, analytic approach, and key findings related to NSSI. Data extraction was conducted by a single reviewer. All characteristics from the included studies can be found in Table 1.

### 2.4. Summarizing and Reporting the Results

Findings were synthesized descriptively and organized thematically according to the ecological level of identified factors, distinguishing between risk and protective factors.

Specifically, identified factors were grouped into conceptual domains (e.g., psychological, family-level, and community/structural factors), allowing a structured representation of how NSSI-related risk and protection are distributed across multiple ecological levels within SGM individuals. Both risk and protective factors identified in the included studies are presented in Table 2.

## 3. Results

### 3.1. PRISMA Flow Diagram

As shown in the PRISMA flowchart, the database search yielded 1029 records. After removing 351 duplicates, 678 records underwent title and abstract screening. Forty-nine reports were sought for full-text retrieval and were assessed for eligibility. Forty-three met the inclusion criteria and were included in the final synthesis. All included studies were primary research reports; no secondary evidence syntheses or systematic reviews were included in the final data extraction to maintain independence of the findings.

### 3.2. Spatiotemporal Distribution and Study Characteristics

Studies were published between 2014 and 2026, with the vast majority being published in 2018 or later (*n* = 36; 83.7%). Most research was conducted in the United States (*n* = 23; 53.5%), followed by China (*n* = 6; 14.0%), and the United Kingdom (*n* = 5; 11.6%). Three studies (7.0%) utilized multinational or international samples. The remaining research was conducted in Australia, Hungary, Iceland, New Zealand, South Korea, and Turkey (*n* = 1 each; 2.3% each). To ensure independence of the findings and prevent double-counting, multinational studies were treated as a distinct category and were not disaggregated into individual country counts within the frequency distribution.

Regarding study designs, the sample was predominantly composed of cross-sectional studies (*n* = 35; 81.4%), while the remainder included longitudinal designs (*n* = 3; 7.0%), qualitative methodologies (*n* = 3; 7.0%), and one study each using Ecological Momentary Assessment and quasi-experimental designs (2.3% each).

Sample sizes ranged from *N* = 15 in qualitative interview-based studies to *N* = 64,651 in large-scale school-based studies. The main study design was cross-sectional (*n* = 34; 79.1%), followed by longitudinal designs (*n* = 3; 7.0%), including prospective cohort and micro-longitudinal designs. Three studies (7.0%) used qualitative interview-based methodologies. Ecological Momentary Assessment and quasi-experiment designs were used in one study each (2.3%). Substantial variation was observed in the measurement of NSSI. Single-item self-report measures were used in 17 studies (39.5%), multi-item validated instruments (e.g., ISAS, DSHI, FASM) in 20 studies (46.5%), and interview-based assessments in six studies (14.0%).

Regarding age groups, 17 (39.5%) studies included adolescents (≤18 years), 15 (34.9%) included young adults (18–30 years), and 6 (14.0%) included adults (>30 years). The remaining studies (*n* = 5; 11.6%) utilized mixed samples or covered wide age ranges from adolescence to late adulthood. As for the study population, 16 studies (37.2%) focused on SGM individuals, 17 (39.5%) on transgender and gender-diverse individuals, and 10 (23.3%) on combined sexual and gender minority samples. A small number of studies focused on specific subgroups, including bisexual individuals (*n* = 3), asexual individuals (*n* = 1), and men who have sex with men (*n* = 1).

### 3.3. Risk Factors for NSSI

#### 3.3.1. The Risk Factors for NSSI Within an Ecological Framework

To synthesize risk correlates across heterogeneous designs, measures, and SGM subgroups, we organized extracted determinants of NSSI using a minority stress–informed socioecological framework. Socioecological theory (Bronfenbrenner, 1979; McLeroy et al., 1988) conceptualizes health-related behaviors as shaped by interacting individual, relational, and structural systems, while minority stress theory (Meyer, 2003) specifies that sexual and gender minority health disparities emerge through distal stressors (e.g., discrimination, victimization, structural stigma) and proximal processes (e.g., internalized stigma, expectations of rejection). Consistent with these foundations, risk factors identified in the included studies were charted into pragmatic analytic domains reflecting the levels most frequently operationalized in empirical literature. Because most studies examined determinants spanning multiple levels, domain counts are not mutually exclusive.

#### 3.3.2. Psychological and Individual-Level Risk Factors: A Minority Stress Synthesis

Twenty studies (46.5%) identified psychological or intrapersonal factors associated with NSSI. To better reflect the causal logic of minority stress theory and Hatzenbuehler’s (2009) psychological mediation framework, these factors were synthesized into distal stressors, proximal processes, general vulnerabilities, and behavior-specific mechanisms.

##### Distal Minority Stressors

Distal stressors included objective external events that create a hostile environment, such as discrimination, harassment, school-based bullying, and physical or sexual violence (Angoff et al., 2021; Li et al., 2019; McDowell et al., 2019; Staples et al., 2018; Taliaferro et al., 2019). These external experiences were consistently associated with higher NSSI risk across the included studies.

##### Proximal Minority Stress Processes

Proximal processes involved internalized responses to distal stress, including internalized homonegativity or transnegativity, identity concealment, chronic expectations of rejection, and gender dysphoria (Chen et al., 2022; Coleman et al., 2025; Hird et al., 2025; Staples et al., 2018; Ünsal et al., 2025; Yuan et al., 2024). These processes represent the subjective experience of stigma that precedes clinical distress.

##### General Psychological Vulnerabilities

General vulnerabilities were identified as “downstream” consequences or mediators through which minority stress impacts health. These included depressive symptoms, anxiety, psychological distress, and broader psychopathology (Yazkan Akgül et al., 2026; Chen et al., 2022; Claes et al., 2015; Davey et al., 2016; Muehlenkamp & Nagy, 2025; Ramsay-Patel et al., 2026; Speer et al., 2022; Watson & Tatnell, 2022). Cognitive-affective styles, such as self-criticism, rumination, perceived burdensomeness, and negative body image, were also identified as key pathways to NSSI (Chen et al., 2022; Coleman et al., 2025; Dumas & Pepper, 2023; Fraser et al., 2018; D. M. Smith et al., 2020).

#### 3.3.3. Family-Level Risk Factors

Nine studies (20.9%) identified family-related risk factors for NSSI. These included family maladjustment, lower family cohesion and adaptability, and difficulties in parent–child relationships and communication (Katz-Wise et al., 2018; D. M. Smith et al., 2020; Ying et al., 2025). Lower perceived parental monitoring, reduced family support, and family strain were also associated with NSSI (Benau et al., 2017; Ross-Reed et al., 2019; D. M. Smith et al., 2020). Additionally, adverse childhood experiences were identified as risk factors, including childhood abuse, neglect, and early family adversity (Cao et al., 2023; Guo et al., 2025; Kuhlemeier et al., 2023; Li et al., 2019).

#### 3.3.4. School and Peer-Level Risk Factors

Ten studies (23.3%) identified school- and peer-level risk factors associated with NSSI. Bullying, peer victimization, harassment, and teasing related to sexual orientation or gender expression emerged as consistent factors (Angoff et al., 2021; Kuhlemeier et al., 2023; McDowell et al., 2019; Li et al., 2019; Ross-Reed et al., 2019; Taliaferro et al., 2018, 2019, 2024). Other school-related stressors included negative school environments and academic stress (Coleman et al., 2025; Parodi et al., 2022).

#### 3.3.5. Community-Level Risk Factors

Five studies (11.6%) identified community-level risk factors, including social isolation, reduced sense of belonging, broader community marginalization and lack of safety (Arcelus et al., 2016; Claes et al., 2015; Dumas & Pepper, 2023; Guo et al., 2025; Parodi et al., 2022). Qualitative evidence also highlighted identity invalidation, community stigma, and social disconnection—particularly among bisexual and other marginalized subgroups—as relevant risk contexts (Dumas & Pepper, 2023).

#### 3.3.6. Structural and Societal-Level Risk Factors

Fourteen studies (32.6%) identified macro-level structural and societal influences on NSSI risk. These include broad systems of oppression such as heterosexism, transphobia, biphobia, and structural stigma arising from institutional policies or the lack of legal protections (Chen et al., 2022; Dumas & Pepper, 2023; Guo et al., 2025; K. B. Jackman et al., 2018; K. Jackman et al., 2018; Muehlenkamp et al., 2015; Reisner et al., 2014; Sigurvinsdottir et al., 2024; Speer et al., 2022; Staples et al., 2018; Ünsal et al., 2025; Yim et al., 2025; Yuan et al., 2024). Specifically, restrictive sociopolitical climates and systemic discrimination were found to amplify the psychological burden on SGM individuals, providing the foundational context for the distal and proximal stress processes described at the individual level (Ünsal et al., 2025; Staples et al., 2018).

### 3.4. Protective Factors of NSSI

#### 3.4.1. Direct Protective Factors

Direct protective factors were associated with lower NSSI outcomes regardless of risk level. Psychological resilience was significantly correlated with a reduced history of NSSI and better overall mental health outcomes across the included samples (Davey et al., 2016; Watson & Tatnell, 2022). Similarly, psychological empowerment, characterized by a sense of personal agency and self-efficacy, directly predicted reduced NSSI frequency and lower suicide risk among SGM young adults (Liang & Chen, 2024; Muehlenkamp & Nagy, 2025). Positive self-regard, specifically self-esteem and body satisfaction, was also identified as a direct correlate of reduced NSSI engagement (Arcelus et al., 2016; Sigurvinsdottir et al., 2024).

#### 3.4.2. Buffering Protective Factors

Buffering factors refer to variables that interact with risk factors to moderate and attenuate their impact on NSSI behavior. Perceived social support from parents, peers, and significant others emerged as a primary moderator of the relationship between minority stress and NSSI (Arcelus et al., 2016; Guo et al., 2025; Sigurvinsdottir et al., 2024). Parental connectedness and school safety were found to moderate the link between bullying victimization and NSSI, particularly among gender minority youth (Taliaferro et al., 2018; Kuhlemeier et al., 2023; Ross-Reed et al., 2019; Parodi et al., 2022). In specific cultural contexts, reciprocal filial piety functioned as a buffer between current NSSI and future suicide risk (Ying et al., 2025).

#### 3.4.3. Risk-Reducing Factors (Absence of Risk)

Several factors associated with lower NSSI risk represented the absence of an identified stressor rather than an active protective process. Lower scores on internalized transnegativity or homonegativity were consistently associated with lower NSSI rates across studies (K. Jackman et al., 2018; Staples et al., 2018). Additionally, higher identity-appearance congruence, indicating lower levels of gender dysphoria, was associated with a decreased likelihood of past-year NSSI (Dunlop et al., 2022; Hird et al., 2025).

### 3.5. Heterogeneity of Risk and Protective Factors Across SGM Subgroups

While SGM populations are often aggregated, the included studies highlight distinct risk and protective profiles across subgroups. Synthesis of these data reveals a “stepwise” pattern of risk, with gender minority (GM) individuals often reporting higher levels of psychological distress and lifetime NSSI compared to cisgender sexual minority (CSM) peers (Ramsay-Patel et al., 2026; Ünsal et al., 2025).

#### 3.5.1. Bisexual and Pansexual Individuals: The Impact of Binegativity

Bisexual individuals emerged as a high-risk subgroup, often exhibiting higher NSSI rates than monosexual gay or lesbian counterparts (Coleman et al., 2025; Dumas & Pepper, 2023). A unique distal stressor for this group is binegativity—stigma and invalidation stemming from both heterosexual and lesbian/gay communities (Coleman et al., 2025; Dumas & Pepper, 2023). This “double marginalization” contributes to social isolation and self-loathing, which are identified as primary drivers for self-punitive NSSI (Dumas & Pepper, 2023).

#### 3.5.2. Transgender and Gender-Diverse (TGD) Individuals: Gender Dysphoria

For TGD populations, gender dysphoria functions as a potent, specific stressor not applicable to cisgender sexual minorities (Yazkan Akgül et al., 2026; Hird et al., 2025). Distress from the incongruence between identity and birth-assigned sex—often exacerbated by transphobic victimization—leads many TGD youth to use NSSI to cope with or “punish” gendered body parts (Hird et al., 2025). Furthermore, transgender men consistently reported higher lifetime NSSI prevalence and more frequent self-cutting than transgender women across several samples (Ünsal et al., 2025).

#### 3.5.3. Asexual Youth: Erasure and Invisibility

Asexual youth face distinct minority stress characterized by identity erasure and invisibility (Liang & Chen, 2024). Societal normativity around allosexuality often leads to asexual identity being dismissed as a clinical abnormality, invalidating the individual’s experience (Liang & Chen, 2024). While some evidence suggests asexual individuals may report lower lifetime NSSI risk than other LGB subgroups, the intersection of asexuality with transgender identity significantly escalates risk (Liang & Chen, 2024).

### 3.6. Functional Heterogeneity of NSSI

A cross-synthesis of findings from the subset of studies assessing motivations suggests a systematic link between specific stress processes and NSSI functions. Proximal minority stressors, such as internalized transnegativity and self-criticism, as well as general psychological vulnerabilities (e.g., depressive symptoms), were robustly associated with intrapersonal functions, primarily affect regulation and self-punishment (Chen et al., 2022; Ünsal et al., 2025; Yim et al., 2025). Conversely, interpersonal functions, including social signaling and ‘cries for help ‘, emerged more frequently in the context of distal stressors, such as peer victimization, school-based bullying, and parental rejection (Peters et al., 2020; Ying et al., 2025; Yuan et al., 2024). In these cases, NSSI may function as a costly social signal to communicate distress when support systems are perceived as invalidating (Yuan et al., 2024).

## 4. Discussion

This scoping review synthesized evidence from 43 studies to map the multi-level risk and protective factors associated with NSSI in SGM populations. While previous meta-analyses have primarily established prevalence estimates and broad correlates, our findings provide a structured socioecological mapping of how structural stressors, interpersonal adversity, and intrapersonal vulnerabilities may co-occur and interact in shaping NSSI risk.

### 4.1. The Recursive Cycle of Risk: Minority Stress and Psychological Vulnerability

A core contribution of this review is the identification of the potentially recursive nature of risk in SGM individuals. Consistent with the psychological mediation framework (Hatzenbuehler, 2009), our synthesis suggests that NSSI is frequently associated with a co-occurrence of distal structural stressors—such as discrimination and victimization—and proximal psychological vulnerabilities, including internalized stigma, self-criticism, emotional distress, and emotion dysregulation. As demonstrated by Chen et al. (2022) and D. M. Smith et al. (2022), it is the subjective processing of stigma through maladaptive cognitive and affective styles, such as self-criticism and heightened emotional reactivity, that often triggers and maintains the self-injurious cycle.

Our synthesis further elucidates the functional specificity of these risk pathways. While proximal minority stress (e.g., internalized stigma) appears to drive intrapersonal functions by depleting internal coping resources (Chen et al., 2022; Yim et al., 2025), distal stressors (e.g., victimization) may uniquely trigger interpersonal functions as individuals seek to communicate distress in hostile social environments (Ying et al., 2025; Yuan et al., 2024). This suggests that clinical targets should be differentiated: interventions focusing on internal emotional regulation and self-compassion may be most effective for those driven by proximal stress, whereas those targeting social signaling functions may require a greater focus on environmental safety and interpersonal support systems (Peters et al., 2020; Taliaferro et al., 2024). While psychological distress frequently precedes engagement in NSSI, theoretical models and emerging longitudinal evidence suggest that this relationship may be bidirectional, potentially creating a recursive cycle of risk (Ramsay-Patel et al., 2026). For instance, prospective data indicate that NSSI may exacerbate psychological symptoms over time through mechanisms of internalized shame and social rejection (Zhu et al., 2020), which may further erode the protective social fabric available to SGM individuals (Peters et al., 2020). Within LGBTQ+ populations, this self-perpetuating loop may be especially pronounced, as NSSI has been shown to increase feelings of alienation and expose individuals to further discrimination (Burke et al., 2019; Ramsay-Patel et al., 2026). However, as most of the current evidence base remains cross-sectional (e.g., Shepherd et al., 2024), these bidirectional pathways should be interpreted as theoretical propositions requiring more robust longitudinal testing to establish definitive temporal ordering.

### 4.2. Shifting Paradigms: From Risk-Reduction to Protection-Promotion

Beyond risk reduction, this review highlights the need for a more granular understanding of protective and resilience-promoting processes. Consistent with the resiliency framework, our findings distinguish between direct protective factors, such as resilience and psychological empowerment, which are associated with lower NSSI regardless of minority stress levels (Davey et al., 2016; Muehlenkamp & Nagy, 2025), and buffering factors, like family connectedness and school safety, which specifically attenuate the impact of stressors such as bullying (Parodi et al., 2022; Taliaferro et al., 2018). In contrast, factors such as lower internalized stigma or higher identity congruence may be more accurately conceptualized as risk-reducing factors—representing the absence or reduced internalization of minority stress rather than active protective mechanisms (Staples et al., 2018).

A critical finding in this synthesis is the context-dependent nature of protective factors, exemplified by the ‘concealment paradox.’ While identity concealment is typically classified as a proximal minority stressor associated with poor mental health, Chen et al. (2022) identified a short-term protective effect, where concealment may temporarily shield individuals from distal discrimination in hostile environments. However, this protection is ‘dual-edged’; while it may alleviate the immediate risk of victimization, it may simultaneously increase the internal psychological burden through self-criticism and ego depletion (Chen et al., 2022; Liang & Chen, 2024). Similarly, ‘outness’ or identity disclosure is not inherently protective; its effect is contingent upon the social climate. In affirming contexts, disclosure facilitates access to social resources and authentic relationships (Shepherd et al., 2024), but in hostile settings, it escalates exposure to violence and discrimination, thereby increasing NSSI risk (Liang & Chen, 2024; Shepherd et al., 2024). This tension underscores that for SGM individuals, the ‘protective’ value of a factor is inextricably linked to the structural safety and affirming quality of their environment.

### 4.3. Subgroup Heterogeneity and Developmental Salience

Beyond the subgroup-specific risk profiles identified in the Results (Section 3.5), our synthesis highlights adolescence and young adulthood as a pivotal developmental window for NSSI engagement. This period is uniquely characterized by the dual tasks of identity exploration and consolidation, where SGM youth must navigate the “coming out” process, a series of identity disclosures that are often emotionally tumultuous and can lead to interpersonal rejection or a stressful need for identity concealment (Shepherd et al., 2024; Taliaferro et al., 2024).

Furthermore, the school environment serves as a primary context where heteronormative expectations and peer hierarchies are most aggressively policed. Because adolescents are uniquely dependent on these social systems for belonging, experiences of bullying in this setting become particularly acute, functioning as primary drivers for NSSI onset (Kuhlemeier et al., 2023; Parodi et al., 2022; Taliaferro et al., 2018). Notably, our review identifies a significant gap in evidence regarding later adulthood. In older cohorts, the focus of minority stress likely shifts from school-based victimization toward structural stressors, such as employment discrimination and lack of legal recognition, which require further longitudinal exploration (Cao et al., 2023; McDowell et al., 2019).

### 4.4. Limitations and Future Directions

Despite the depth of this synthesis, several methodological constraints remain. The literature is predominantly cross-sectional, which limits the ability to disentangle the temporal sequence of the recursive risk cycle definitively. The temporal direction of the identified protective factors remains a significant area of uncertainty. Due to the cross-sectional nature of the data, it is unclear whether family and school connectedness proactively reduce NSSI risk or whether youth with lower self-injurious tendencies are better able to foster and maintain supportive relationships (Ross-Reed et al., 2019; Taliaferro et al., 2019). This possibility of bidirectionality must be foregrounded, as NSSI itself can impair interpersonal functioning and lead to social withdrawal, which may further erode the protective social fabric available to these individuals (Peters et al., 2020; Ramsay-Patel et al., 2026). Additionally, although several studies examined NSSI motivations, there is significant heterogeneity in how NSSI functions are assessed and reported across the literature. This lack of standardized functional analysis across the full sample prevents a definitive mapping of how specific risk factors (e.g., victimization vs. internalized stigma) differentially predict distinct NSSI functions, representing a critical gap for future research. Additionally, although several studies examined NSSI motivations, there is significant heterogeneity in how NSSI functions are assessed and reported across the literature. This lack of standardized functional analysis across the full sample prevents a definitive mapping of how specific risk factors (e.g., victimization vs. internalized stigma) predict distinct NSSI functions, representing a critical gap for future research. Furthermore, it must be noted that only a subset of the included studies (*n* = 11) provided sufficient data to link specific risk factors to distinct NSSI functions. The lack of systematic cross-tabulation across the full evidence base remains a significant limitation, preventing a definitive mapping of how various socioecological determinants predict functional heterogeneity across all SGM subgroups and developmental stages.

Additionally, the decision to restrict inclusion to peer-reviewed studies and thereby exclude grey literature such as dissertations, conference proceedings, and preprints must be acknowledged as a potential source of publication bias. By focusing exclusively on published research, this review may systematically overrepresent studies with significant or positive findings, potentially underrepresenting the full range of evidence, including null results or unexpected findings that are less frequently published in core academic journals. Finally, although the search was conducted across three major multidisciplinary databases, the exclusion of more specialized platforms (e.g., PsycINFO) must be acknowledged as a limitation. While the retrieval of 43 included studies suggests a substantial mapping of the existing evidence, it remains possible that a small number of studies indexed exclusively in specialized databases were not captured.

Future research should prioritize longitudinal designs to clarify the directionality and dynamic interaction between minority stress and NSSI. Furthermore, as most of the studies were conducted in the United States, there is a need for more culturally and geographically diverse research to understand how different geopolitical climates influence SGM risk and resilience.

### 4.5. Practical and Policy Implications

The findings suggest that prevention and intervention efforts should address both individual-level mechanisms and the broader social conditions that shape NSSI risk among SGM individuals. At the individual level, clinicians may consider SGM-affirmative cognitive-behavioral approaches to address negative beliefs, stigma-related cognitions, and maladaptive cognitive-affective styles (Chen et al., 2022; Ünsal et al., 2025); and adapted Dialectical Behavior Therapy (DBT) to specifically address emotion dysregulation and distress tolerance within a minority stress framework (Peters et al., 2020; E. R. Smith & Perrin, 2017; Ünsal et al., 2025). Specifically, given that self-criticism, emotion dysregulation, internalized stigma, and concealment-related cognitions emerged as the most consistently identified modifiable mechanisms across the synthesis (e.g., Chen et al., 2022; Fraser et al., 2018; D. M. Smith et al., 2022; Staples et al., 2018), interventions should prioritize these specific targets to effectively disrupt the NSSI cycle, rather than focusing solely on general distress reduction.

At the interpersonal and structural levels, efforts should prioritize psychoeducation for families to prevent invalidating or stigmatizing responses to NSSI disclosure (Taliaferro et al., 2024; Yuan et al., 2024) and the institutionalization of school safety. This includes the implementation of inclusive curricula that foster gender and sexuality literacy and the active support of Gender-Sexuality Alliances (GSAs), which have been identified as vital for promoting student self-efficacy and school connectedness (K. Jackman et al., 2018; Parodi et al., 2022; Taliaferro et al., 2019).

Finally, at the structural level, the findings support the importance of policies that reduce discrimination and increase access to affirming services. Anti-discrimination protections, gender-affirming healthcare access, inclusive mental health services, and legal recognition of gender diversity may help reduce the structural stigma that contributes to proximal stress processes and psychological vulnerability (Cao et al., 2023; McDowell et al., 2019; Staples et al., 2018; Ünsal et al., 2025). Because the included studies were largely observational, future intervention and policy evaluation studies are needed to determine which strategies most effectively reduce NSSI risk and promote resilience in SGM populations.

## 5. Conclusions

The evidence synthesized in this review suggests that NSSI among SGM populations is associated with the co-occurrence of minority stress-related adversity and psychological vulnerability, rather than with any single set of factors in isolation. Structural and interpersonal stressors, such as discrimination and victimization, seem to intersect with intrapersonal processes, including emotional distress, emotion dysregulation, and cognitive-affective vulnerabilities, in potentially shaping NSSI risk. Furthermore, this synthesis underscores the clinical importance of subgroup heterogeneity, suggesting that gender minority individuals, bisexual individuals, and other multiply marginalized subgroups may experience elevated vulnerability, often in relation to group-specific stressors such as gender dysphoria, transphobic victimization, binegativity, and identity invalidation. In contrast, the comparative lack of research on protective factors remains a critical gap, which may limit a more comprehensive understanding of resilience processes in this population. Existing evidence suggests that family connectedness, peer support, school safety, identity affirmation, resilience, and psychological empowerment may play protective or buffering roles, although their effects appear to depend strongly on developmental stage and social context.

Overall, these findings lend support to the utility of a multilevel perspective, consistent with minority stress and socioecological frameworks, in conceptualizing NSSI among SGM individuals. However, given the predominantly cross-sectional nature of the sampled literature, these associations should be interpreted with caution, as longitudinal research remains essential to establish definitive causal pathways and temporal directionality. Future research should also prioritize culturally diverse, intervention-oriented, and subgroup-sensitive designs to clarify modifiable mechanisms and inform affirming, strengths-based prevention strategies.

## Figures and Tables

**Table 1:** Characteristics of the 43 Primary Studies Included in the Synthesis.

Study (Author, Year)	Country	Sample Size	SGM Subgroup	Study Design	Age Range	NSSI Measure
(Yazkan Akgül et al., 2026)	Turkey	92	Transgender and Non-binary	Cross-sectional	10–17 years	ISAS
(Angoff et al., 2021)	USA	49,425	SGM Youth (LGB & Trans)	Cross-sectional	~13–18 years	Single-item
(Arcelus et al., 2016)	UK	268	Transgender Youth	Cross-sectional	18–25 years	SIQ-TR
(Benau et al., 2017)	USA	1352	Sexual Minority	Cross-sectional	18–25 years	FAFSI
(Cao et al., 2023)	China	971	Transgender (MTF/FTM)	Cross-sectional	M ≈ 24.6 years	FASM
(Chen et al., 2022)	China	666	Sexual Minority (Gay/Bi)	Cross-sectional	18–68 years	ISAS
(Claes et al., 2015)	UK	155	Transgender Adults	Cross-sectional	17–77 years	SIQ
(Coleman et al., 2025)	UK	143	Bisexual Youth	Micro-longitudinal	13–18 years	Weekly self-report
(Davey et al., 2016)	UK	97	Transgender Adults	Cross-sectional	M = 36.18 years	ISAS-based
(Dumas & Pepper, 2023)	USA	259	Cisgender Sexual Minorities	Cross-sectional	19–66 years	ISAS
(Dunlop et al., 2022)	UK	15	Bisexual Youth	Qualitative	16–25 years	Interview
(Fraser et al., 2018)	New Zealand	1799	LGB Adolescents	Cross-sectional	13–18 years	DSHI-s
(Guo et al., 2025)	China	64,651	SGM Adolescents	Cross-sectional	M ≈ 15.13 years	FASM
(Hird et al., 2025)	Multinational	923	Transgender Youth	Cross-sectional	17–25 years	ISAS
(K. B. Jackman et al., 2018)	USA	332	Transgender Adults	Cross-sectional	16–87 years	SITBI
(K. Jackman et al., 2018)	USA	18	Transmasculine Spectrum	Qualitative	17–38 years	Interview
(Katz-Wise et al., 2018)	USA	33	TGD Youth	Cross-sectional	13–17 years	Single-item
(Kuhlemeier et al., 2023)	USA	17,811	SGM Youth	Cross-sectional	High school	YRRS-based
(Li et al., 2019)	China	1931	Sexual Minority Adolescents	Cross-sectional	~15–18 years	Single-item
(Liang & Chen, 2024)	Multinational	5574	Asexual Youth	Cross-sectional	13–24 years	Composite
(McDowell et al., 2019)	USA	150	Transmasculine Adults	Cross-sectional	21–50 years	Single-item
(Muehlenkamp et al., 2015)	USA	137	SM College Students	Cross-sectional	18–30 years	ISAS
(Muehlenkamp & Nagy, 2025)	USA	305	LGBTQ+ Young Adults	Cross-sectional	18–25 years	Single-item
(Parodi et al., 2022)	USA	252	TGD Youth	Cross-sectional	14–30 years	SITBI-R
(Peters et al., 2020)	USA	52	SM Adolescents (Inpatient)	Cross-sectional	12–18 years	ISAS
(Ramsay-Patel et al., 2026)	Multinational	1475	LGBTQ+ Young Adults	Longitudinal	18–29 years	SITBI-based
(Reisner et al., 2014)	USA	3131	LGBQ Youth	Cross-sectional	14–18 years	Single-item
(Ross-Reed et al., 2019)	USA	12,687	Gender Minority Youth	Cross-sectional	Grades 9–12	Single-item
(Shepherd et al., 2024)	USA	792	SM (Gay/Bi+)	Longitudinal	18–30 years	SITBI
(Sigurvinsdottir et al., 2024)	Iceland	8291	Sexual Minority Youth	Cross-sectional	16–19 years	Single-item
(E. R. Smith & Perrin, 2017)	USA	239	Sexual Minority Adults	Cross-sectional	18–66 years	Single-item
(D. M. Smith et al., 2020)	USA	252	SGM Adolescents	Longitudinal	14–15 years	SITBI-based
(D. M. Smith et al., 2022)	USA	330	SGM Adolescents	Quasi-experimental	14–15 years	Self-report
(Speer et al., 2022)	USA	10,330	LGBTQ Adolescents	Cross-sectional	16–25 years	ISAS
(Staples et al., 2018)	USA	237	Transgender Adults	Cross-sectional	18–44 years	DSHI
(Taliaferro et al., 2018)	USA	2168	Transgender/GNC Youth	Cross-sectional	Grades 9/11	Single-item
(Taliaferro et al., 2019)	USA	1635	Transgender/GNC Youth	Cross-sectional	Grades 9/11	Single-item
(Taliaferro et al., 2024)	USA	58	Sexual Minority Women	Qualitative	14–25 years	Interview
(Ünsal et al., 2025)	Hungary	202	TGD Adults	Cross-sectional	18–74 years	ISAS
(Watson & Tatnell, 2022)	Australia	330	LGBTQIA+ Individuals	Cross-sectional	18–66 years	ISAS
(Yim et al., 2025)	S. Korea	431	Sexual Minority	Cross-sectional	19–29 years	ISAS
(Ying et al., 2025)	China	335	Gay Men	Cross-sectional	18–25 years	Single-item
(Yuan et al., 2024)	China	2612	MSM	Cross-sectional	18–68 years	ISAS

**Table 2:** Findings from each study cited.

Study (Author, Year)	Risk Factors	Protective Factors
(Angoff et al., 2021)	Bullying (school, online, orientation-based); depression; substance use; dating violence; AFAB	Supportive school adults; perceived school structure; higher grade level
(Arcelus et al., 2016)	Higher psychological distress; lower self-esteem; body dissatisfaction; low social support	Higher social support; self-esteem; body satisfaction
(Benau et al., 2017)	Low parental monitoring	Higher parental monitoring
(Cao et al., 2023)	Childhood abuse (emotional, physical, sexual); neglect; emotional dysregulation	Not directly tested
(Chen et al., 2022)	Sexual minority stigma; self-criticism; depression; concealment (indirect effects);	Sexual orientation concealment (short-term protective effect)
(Claes et al., 2015)	Psychological symptoms; victimization; interpersonal difficulties; low perceived social support	Not directly tested
(Coleman et al., 2025)	Rumination; binegativity (internalised biphobia)	Not directly tested
(Davey et al., 2016)	Depression; anxiety; stress; low resilience; concealment	Higher resilience; identity openness
(Dumas & Pepper, 2023)	Biphobic stigma; rejection; marginalisation; social disconnection; internalised stigma; emotion dysregulation; lack of coping; intersectional discrimination	Identity affirmation; bisexual community belonging
(Dunlop et al., 2022)	Perceived stigma; gender dysphoria; minority stress;	Transgender congruence
(Fraser et al., 2018)	Sexuality-related stress; emotion dysregulation	Emotion regulation capacities
(Guo et al., 2025)	Adverse childhood experiences; cumulative adversity; gender nonconformity	Social support
(Hird et al., 2025)	Transphobic experiences; body dissatisfaction; body surveillance; gender dysphoria	Not directly tested
(K. B. Jackman et al., 2018)	Gender dysphoria; stigma; minority stress	Not directly tested
(K. Jackman et al., 2018)	Internalized transnegativity; discrimination; victimization	Lower internalized transnegativity
(Katz-Wise et al., 2018)	Poor family communication; low family satisfaction; stigma; victimization	Family communication; family satisfaction
(Kuhlemeier et al., 2023)	Violence victimization; low family, school, and peer support	School connectedness; caring adults
(Li et al., 2019)	Adverse childhood experiences; bullying; academic stress; substance use	Not directly tested
(Liang & Chen, 2024)	Minority stress; discrimination; violence	Psychological empowerment
(McDowell et al., 2019)	Mental health problems; abuse; relationship violence; bullying; substance use	Relationship support
(Muehlenkamp et al., 2015)	Sexual minority status; depression; hopelessness	Not directly tested
(Muehlenkamp & Nagy, 2025)	Psychological distress; minority stress; younger age	Psychological empowerment
(Parodi et al., 2022)	Negative school environment; lack of safety	School connectedness
(Peters et al., 2020)	Psychiatric symptoms; comorbidity	Not directly tested
(Ramsay-Patel et al., 2026)	Psychological distress	Not directly tested
(Reisner et al., 2014)	Minority stress; abuse; victimization	Family support; resilience resources
(Ross-Reed et al., 2019)	Violence victimization; low family, school, and peer support	Family, school, and peer support
(Shepherd et al., 2024)	Discrimination; depressive symptoms; identity disclosure (indirect via discrimination)	Identity disclosure in affirming contexts
(Sigurvinsdottir et al., 2024)	Minority stress; psychological mediators	Not directly tested
(E. R. Smith & Perrin, 2017)	Heterosexism; anxiety; depression; low life satisfaction	Better mental health; life satisfaction
(D. M. Smith et al., 2020)	Abuse; discrimination; self-criticism; family strain; poor body image; prior suicidality	Family support
(D. M. Smith et al., 2022)	Discrimination; emotional reactivity	Not directly tested
(Speer et al., 2022)	Intersectional minority stress; psychological distress	Not directly tested
(Staples et al., 2018)	Distal and proximal minority stress; internalized transphobia	Lower internalized stigma
(Taliaferro et al., 2018)	Bullying; victimization	School connectedness; adult support
(Taliaferro et al., 2019)	Mental health problems; abuse; bullying; substance use	Family and school connectedness
(Taliaferro et al., 2024)	Stigma; disclosure-related stress; invalidation	Supportive responses (peers, professionals)
(Ünsal et al., 2025)	Gender minority stressors; internalized stigma	Not directly tested
(Watson & Tatnell, 2022)	Low resilience; psychological distress	Resilience
(Yazkan Akgül et al., 2026)	Depressive symptoms; emotional/behavioral problems; psychiatric comorbidity (e.g., MDD, ADHD)	Not directly tested
(Yim et al., 2025)	Sexual minority stigma; intrapersonal vulnerabilities; depression	Cognitive reframing
(Ying et al., 2025)	Cultural stigma; negative parental expectations	Reciprocal filial piety
(Yuan et al., 2024)	Perceived stigma; internalized stigma	Not directly tested

## Data Availability

The data supporting the findings of this study are available within the article and its Supplementary Materials (Appendix A, Table 1 and Table 2). Additional data extraction materials are available from the corresponding author upon reasonable request.

## Supplementary Materials

The following supporting information can be downloaded at: https://www.mdpi.com/article/10.3390/ejihpe16070086/s1, Table S1: PRISMA Checklist.

## Abbreviations

The following abbreviations are used in this manuscript:

Abbreviation	Meaning
ACEs	Adverse Childhood Experiences
AFAB	Assigned Female at Birth
C-SSRS	Columbia-Suicide Severity Rating Scale
CDC	Centers for Disease Control and Prevention
CESD-R	Center for Epidemiologic Studies Depression Scale–Revised
DASI	Deliberate Self-Harm/Self-Injury Assessment item
DSHI	Deliberate Self-Harm Inventory
DSHI-s	Deliberate Self-Harm Inventory–short form
EMA	Ecological Momentary Assessment
FAFSI	Functions and Addictive Features of Self-Injury Scale
FASM	Functional Assessment of Self-Mutilation
ISAS	Inventory of Statements About Self-Injury
LDS	The Church of Jesus Christ of Latter-day Saints
LGBT	Lesbian, Gay, Bisexual, and Transgender
LGBTQ+	Lesbian, Gay, Bisexual, Transgender, Queer/Questioning, and other sexual and gender minority identities
M	Mean
MDD	Major Depressive Disorder
NSSI	Non-Suicidal Self-Injury
OSI	Ottawa Self-Injury Inventory
PRISMA	Preferred Reporting Items for Systematic Reviews and Meta-Analyses
PTSD	Post-Traumatic Stress Disorder
RSE	Rosenberg Self-Esteem Scale
SD	Standard Deviation
SEM	Structural Equation Modeling
SGM	Sexual and Gender Minority
SIQ	Self-Injury Questionnaire
SIQ-TR	Self-Injury Questionnaire–Treatment Related
SITB	Self-Injurious Thoughts and Behaviors
SITBI	Self-Injurious Thoughts and Behaviors Interview
SITBI-R	Self-Injurious Thoughts and Behaviors Interview–Revised
SMS	Sexual Minority Stigma
TGNC	Transgender and Gender-Nonconforming
UK	United Kingdom
USA	United States of America
YRRS	Youth Risk and Resiliency Survey
YSR	Youth Self-Report

## Appendix A

**Table A1:** Search strategies used across databases.

Data Base	Search Strategy
**Scopus**	TITLE-ABS-KEY ((LGBTQ* OR LGB* OR “sexual minorit*” OR “gender minorit*” OR lesbian* OR gay OR bisexual* OR transgender* OR queer OR “gender diverse” OR nonbinary OR “non-binary” OR TGNC) AND (“self injur*” OR “self harm*” OR “non-suicidal self-injury” OR “nonsuicidal self injury” OR “self-injurious behavior” OR “self-injurious behaviour” OR NSSI OR “deliberate self-harm”) AND (“risk factor*” OR “protective factor*” OR predictor* OR correlat* OR associat* OR mediator* OR moderator* OR stigma OR discrimination OR “minority stress” OR “structural stigma” OR marginali* OR “social exclusion” OR victimi* OR harassment OR family OR “family support” OR school* OR “school connectedness” OR bullying OR “peer victimization” OR healthcare OR policy OR community OR “social support” OR resilience OR coping OR “emotion regulation”))
**PubMed**	LGBTQ*[Title/Abstract] OR LGB*[Title/Abstract] OR “sexual minority”[Title/Abstract] OR “gender minority”[Title/Abstract] OR lesbian*[Title/Abstract] OR gay[Title/Abstract] OR bisexual*[Title/Abstract] OR transgender*[Title/Abstract] OR queer[Title/Abstract] OR “gender diverse”[Title/Abstract] OR nonbinary[Title/Abstract] OR “non-binary”[Title/Abstract] OR TGNC[Title/Abstract]) AND (“non-suicidal self-injury”[Title/Abstract] OR “nonsuicidal self injury”[Title/Abstract] OR “self injury”[Title/Abstract] OR “self harm”[Title/Abstract] OR “self-injurious behavior”[Title/Abstract] OR “self-injurious behaviour”[Title/Abstract] OR NSSI[Title/Abstract] OR “deliberate self-harm”[Title/Abstract]) AND (“risk factor*”[Title/Abstract] OR “protective factor*”[Title/Abstract] OR predictor*[Title/Abstract] OR correlat*[Title/Abstract] OR associat*[Title/Abstract] OR mediator*[Title/Abstract] OR moderator*[Title/Abstract] OR stigma[Title/Abstract] OR discrimination[Title/Abstract] OR “minority stress”[Title/Abstract] OR “structural stigma”[Title/Abstract] OR marginali*[Title/Abstract] OR “social exclusion”[Title/Abstract] OR victimi*[Title/Abstract] OR harassment[Title/Abstract] OR family[Title/Abstract] OR “family support”[Title/Abstract] OR school*[Title/Abstract] OR “school connectedness”[Title/Abstract] OR bullying[Title/Abstract] OR “peer victimization”[Title/Abstract] OR healthcare[Title/Abstract] OR policy[Title/Abstract] OR community[Title/Abstract] OR “social support”[Title/Abstract] OR resilience[Title/Abstract] OR coping[Title/Abstract] OR “emotion regulation”[Title/Abstract])
**Web of Science**	(LGBTQ* OR LGB* OR “sexual minorit*” OR “gender minorit*” OR lesbian* OR gay OR bisexual* OR transgender* OR queer OR “gender diverse” OR nonbinary OR “non-binary” OR TGNC) AND (“self injur*” OR “self harm*” OR “non-suicidal self-injury” OR “nonsuicidal self injury” OR “self-injurious behavior” OR “self-injurious behaviour” OR NSSI OR “deliberate self-harm”) AND (“risk factor*” OR “protective factor*” OR predictor* OR correlat* OR associat* OR mediator* OR moderator* OR stigma OR discrimination OR “minority stress” OR “structural stigma” OR marginali* OR “social exclusion” OR victimi* OR harassment OR family OR “family support” OR school* OR “school connectedness” OR bullying OR “peer victimization” OR healthcare OR policy OR community OR “social support” OR resilience OR coping OR “emotion regulation”)
